# The first known case of Nocardia wallacei infection in Cyprus

**DOI:** 10.3205/dgkh000585

**Published:** 2025-09-23

**Authors:** Aristos Aristodimou, Zacharias Raptopoulos, Ioannis Hatzimanolis, Paris Zygoulis, Marios Ioannou, Panagiota Maikanti-Charalampous, Pieridou Despo, Yiolanda Herodotou

**Affiliations:** 1 Internal Medicine Department, Limassol General Hospital, State Health Organization Services, Limassol, Cyprus; 2 Pulmonary Medicine Department, Limassol General Hospital, State Health Organization Services, Limassol, Cyprus; 3 Microbiology Department, Nicosia General Hospital, State Health Organization, Nicosia, Cyprus; 4 Limassol, Cyprus

**Keywords:** Nocardia spp., Nocardia wallacei, pulmonary nocardiosis, trimethoprim-sulfamethoxazole (TMP/SMX)

## Abstract

**Aim:**

To present the first known case of *Nocardia (N.) wallacei* infection in Cyprus.

**Method:**

This case report describes the first known *N. wallacei* infection in Cyprus, diagnosed in a 77-year-old man with underlying pulmonary disease, along with the diagnostic workup and treatment.

**Discussion:**

*N. wallacei* is a rare subtype of *Nocardia* spp. with about 14 cases reported in literature. It equally affects immunocompromised as well as immunocompetent patients, causing both pulmonary and extrapulmonary symptoms (CNS, skin). First-line treatment includes trimethoprim/sulfamethoxazole (TMP/SMX), but there are trends of global resistance.

**Conclusion:**

*N. wallacei* has rarely been reported as a cause of infection and this is the first known case reported in Cyprus.

## Zusammenfassung

**Ziel:**

Vorstellung des ersten bekannten Falls einer *Nocardia (N.) wallacei* Infektion auf Zypern.

**Methode:**

Der Fallbericht beschreibt die erste bekannte *N. wallacei* Infektion auf Zypern, die bei einem 77-jährigen Mann mit zugrunde liegender Lungenerkrankung diagnostiziert wurde, sowie die diagnostische Untersuchung und Behandlung.

**Diskussion:**

*N. wallacei* ist ein seltener Subtyp von *Nocardia* spp. Er betrifft immungeschwächte als auch immunkompetente Patienten gleichermaßen und verursacht sowohl pulmonale als auch extrapulmonale Symptome (ZNS, Haut). Die Erstlinienbehandlung umfasst Trimethoprim/Sulfamethoxazol (TMP/SMX); es gibt jedoch Tendenzen zur weltweiten Resistenz.

**Schlussfolgerung:**

*N. wallacei* wurde selten als Infektionsursache gemeldet, und es ist der erste bekannte Fall, der auf Zypern gemeldet wurde.

## Introduction

*Nocardia* is a Gram-positive, aerobic, branching-rod bacterium that is commonly found in soil, water, air, and decomposing vegetation [1]. There are at least 119 recognized *Nocardia* species and of those, about 54 are implicated in human disease [2]. They are described as mostly opportunistic pathogens and tend to cause disease in immunocompromised patients (e.g., diabetics, patients with chronic obstructive pulmonary disease), but they are also seen in immunocompetent patients [3], [4]. Mostly, *Nocardia* spp. are involved in pulmonary disease, but they can also cause cutaneous disease and central nervous system infection, mainly in the form of abscesses [5]. First-line treatment is trimethoprim-sulfamethoxazole (TMP/SMX), but most species are sensitive to other classes of antibiotics as well, and thus a choice is based on the severity of clinical picture and susceptibility of the bacterium cultured. In recent years, the literature has reported that about 41% of *Nocardia* isolates were found to be resistant to TMP-SMX [1]. *N. wallacei* is a subspecies of *N. transvalensis* which, along with 12 other species of *Nocardia*, account for only 9.3% of total Nocardia isolates [2], [6]. In a retrospective study conducted by Lebeaux et al. [7], the prevalence of *N. wallacei* infections in France was estimated to be around 5%. Only a few cases have been described in the literature as it is a rare microorganism, difficult to culture [6]. 

## Case presentation

A 77-year-old ambulatory man with past medical history of hypertension, benign prostate hyperplasia, chronic obstructive pulmonary disease (centrilobular emphysema), pulmonary fibrosis, B12 and folic acid deficiency, visited his pulmonologist due to complaints of unintentional weight loss of about 10 kg over the last 6 months. He denied having fever, night sweats, cough, shortness of breath (at rest and on exertion), fatigue, diarrhea or joint pain. The patient is a retired office worker and did not mention exposure to any sort of agricultural activities (gardening, planting etc.). He has no pets nor other contact with animals.

On clinical examination, the patient was afebrile, hemodynamically stable and oriented to time, place and self. Oxygen saturation was 93% on room air. Heart sounds were normal and regular. Crackles were auscultated bilaterally at the lung bases. The abdomen was soft and non-tender with normal bowel sounds. There was no focal neurological deficit upon examination of the nervous system. No atypical skin lesions were noted. Upon evaluation of the blood tests, a normal white cell count was noted (7820 cells/µL), normocytic anemia with hemoglobin levels of 10.1 g/L and MCV 83.7fL and a normal platelet number (204,000 cells/µL). Clotting times were within the reference range. C-reactive protein was elevated (11.60 mg/L). Urea (42 mg/dL), creatinine (0.88 mg/dL), Na (137 mmol/L), K (4.1 mmol/L), ALP (58U/L), γ-GT (18U/L), AST (20U/L), ALT (10U/L) were all within the respective reference range.

Initially, he was referred for a computed-tomography (CT) scan of the thorax, which revealed diffuse interstitial thickening – the characteristic ‘honeycomb’ appearance, centrilobular emphysema, evidence of widespread bronchiectasis along with hilar (right 1.9 cm x 1.5cm, left 1.5 cm x 0.7 cm) and mediastinal lymphadenopathy (1.1cm x 1.2 cm). Due to these findings, the patient underwent bronchoscopy with endobronchial ultrasound and tissue samples, along with bronchoalveolar lavage (BAL). Tissue biopsy was negative for malignancy, and the BAL culture was positive for *N. wallacei*, resistant to TMP/SMX. 

The patient was referred to the hospital for treatment and was admitted to the pulmonology ward. A CT of the brain was also done and revealed no abscess or lesions that could indicate central nervous system involvement. Treatment with intravenous imipenem/cilastatin 500 mg, four times a day and intravenous amikacin 700 mg, once daily, was commenced based on the antibiogram provided. 

The patient received treatment for 3 weeks as an inpatient and was discharged with moxifloxacin 400 mg once daily with a follow-up appointment. A CT-scan was repeated and showed reduction in the size of mediastinal (6.8 mm x 6.8 mm) and right hilar (1.02 cm x 1.01 cm) lymph nodes as described above. BAL culture, from follow-up bronchoscopy, revealed eradication of the pathogen (normal respiratory flora) and cellular evidence of regressing inflammation. The patient has managed to gain weight. 

## Discussion

*Nocardia* is a Gram-positive bacterium that exists widely in the soil, water and air [2]. It was thought to be an opportunistic pathogen, but in recent years infections have been reported in healthy individuals as well [8]. The case presented above describes a patient with underlying lung disease, who was diagnosed with pulmonary Nocardia infection by TMP/SMX-resistant *N. wallacei.* As reported by Mehta et al. [9] in a retrospective evaluation of 765 *Nocardia* isolates from 1995–2004, resistance to TMP/SMX was around 42%. These isolates were molecularly analyzed and were found to have mutations in the genes encoding for dihydrofolate reductase enzyme and dihydropteroate synthase [9]. *N. wallacei* is considered quite rare, as there have only been around 14 cases reported in various parts of the world, with some affecting immunocompromised patients and some others seen in immunocompetent hosts [10]. In a retrospective analysis of the clinical characteristics of all the *Nocardia* infections recorded in Henan, China from 2017–2023, the authors identified *N. wallacei* in three out of a total of 71 *Nocardia* isolates (prevalence of 4.2%) [11]. These results are consistent with the findings of Hasemi-Shahraki et al. [12], who reported six *N. wallacei* isolates out of 127 samples (5%) in patients with nocardiosis (both pulmonary and extrapulmonary. Epidemiological data from Australia state a percentage of *N. wallacei* (along with other subspecies of *Nocardia*) to be around 8% [13]. It is widely accepted that the first-line treatment for infection by *Nocadia* is TMP/SMX, but data suggest that the percentage of resistant bacteria is rising. Even more so, *N. wallacei* has been reported to be multi-drug resistant, including drugs such as amikacin and clarithromycin [6]. A possible explanation could be the presence of the wallacei-amikacin resistance A (warA) gene that is found in *N. wallacei* [10]. In the case described above, the bacterium was sensitive to amikacin and the carbapenem group, and was treated as such, with the patient ultimately managing to put on weight. 

## Conclusions

Infection by *N. wallacei* has rarely been reported in literature. The patient presented above constitutes the first case of pulmonary nocardiosis by *N. wallacei* in Cyprus. It is also worth mentioning that the percentage of resistance to the first-line treatment, TMP/SMX, is rising. This can be concerning since it could limit the available options for oral and parenteral treatment. 

## Notes

### Competing interests

The authors declare that they have no competing interests.

### Funding

None

### Authors’ ORCIDs

- Aristodimou A:
https://orcid.org/0009-0003-6567-1999
- Raptopoulos Z: https://orcid.org/0009-0008-4798-9203
- Hatzimanolis I: https://orcid.org/0009-0002-9408-7241
- Zygoulis P: https://orcid.org/0009-0004-9828-0430
- Marios Ioannou: https://orcid.org/0009-0005-3384-3965
- Pieridou D: https://orcid.org/0009-0007-8049-2082
- Herodotou Y: https://orcid.org/0009-0002-0918-2181

## References

[1] Saksena R, Rynga D, Rajan S, Gaind R, Dawar R, Sardana R (2020). Fatal pulmonary infection by trimethoprim-sulfamethoxazole resistant Nocardia otitidiscaviarum: report of two cases and review. J Infect Dev Ctries.

[2] Wang H, Zhu Y, Cui Q, Wu W, Li G, Chen D (2022). Epidemiology and antimicrobial resistance profiles of the Nocardia species in China, 2009 to 2021. Microbiol Spectr.

[3] Barry M, AlShehri S, Alguhani A, Barry M, Alhijji A, Binkhamis K (2022). A fatal case of disseminated nocardiosis due to Nocardia otitidiscavium resistant to trimethoprim-sulfamethoxazole: case report and literature review. Ann Clin Microbiol Antimicrob.

[4] Aristodimou A, Nearhou P, Georgian E, Lemesios M (2020). Successful treatment of pulmonary nocardiosis with levofloxacin: case report. Arch Hell Med.

[5] Rathish B, Zito PM (2025). Nocardia.

[6] Ranjan R, Gunasekaran J, Bir R, Kumar U, Gupta RM (2024). First case of Nocardia wallacei from India: a case report and literature review. Cureus.

[7] Lebeaux D, Bergeron E, Berthet J, Djadi-Prat J, Mouniee D, Boiron P (2019). Antibiotic susceptibility testing and species identification of Nocardia isolates: a retrospective analysis of data from a French expert laboratory, 2010–2015. Clin Microbiol Infect.

[8] Cassir N, Mimmion M, Noudel R, Drancourt M, Brouqui P (2013). Sulfonamide resistance in a disseminated infection caused by Nocardia wallacei: a case report. J Med Case Rep.

[9] Mehta H, Weng J, Prater A, Elworth RAL, Han X, Shamoo Y (2018). Pathogenic Nocardia cyriacigeorgica and Nocardia nova evolve to resist trimethoprim-sulfamethoxazole by both expected and unexpected pathways. Antimicrob Agents Chemother.

[10] Chieosilapatham P, Duangsonk K, Kaweewan I, Tongjai S, Kanthawang T (2024). Nocardia wallacei: a rare cause of actinomycetoma in an immunocompetent patient. Eur J Microbiol Immunol (Bp).

[11] Han Y, Cheng M, Li Z, Chen H, Xia S, Zhao Y (2024). Clinical characteristics and drug resistance of Nocardia in Henan, China, 2017–2023. Ann Clin Microbiol Antimicrob.

[12] Hashemi-Shahraki A, Heidarieh P, Bostanabad SZ, Hashemzadeh M, Feizabadi MM, Schraufnagel D (2015). Genetic diversity and antimicrobial susceptibility of Nocardia species among patients with nocardiosis. Sci Rep.

[13] Yeoh K, Globan M, Namo P, Williamson DA, Lea K, Bond K (2022). Identification and antimicrobial susceptibility of referred Nocardia isolates in Victoria, Australia 2009–2019. J Med Microbiol.

